# The Commingled Division of Visual Attention

**DOI:** 10.1371/journal.pone.0130611

**Published:** 2015-06-15

**Authors:** Yuechuan Sun, Sijing Wu, Ian Spence

**Affiliations:** Department of Psychology, University of Toronto, Toronto, Ontario, Canada; Centre de Neuroscience Cognitive, FRANCE

## Abstract

Many critical activities require visual attention to be distributed simultaneously among distinct tasks where the attended foci are not spatially separated. In our two experiments, participants performed a large number of trials where both a primary task (enumeration of spots) and a secondary task (reporting the presence/absence or identity of a distinctive shape) required the division of visual attention. The spots and the shape were commingled spatially and the shape appeared unpredictably on a relatively small fraction of the trials. The secondary task stimulus (the shape) was reported in inverse proportion to the attentional load imposed by the primary task (enumeration of spots). When the shape did appear, performance on the primary task (enumeration) suffered relative to when the shape was absent; both speed and accuracy were compromised. When the secondary task required identification in addition to detection, reaction times increased by about 200 percent. These results are broadly compatible with biased competition models of perceptual processing. An important area of application, where the *commingled* division of visual attention is required, is the augmented reality head-up display (AR-HUD). This innovation has the potential to make operating vehicles safer but our data suggest that there are significant concerns regarding driver distraction.

## Introduction

Divided attention is the simultaneous allocation of attentional resources to two (or more) tasks. The division may cross modalities, as when speaking on a mobile phone while driving a vehicle [1–3]. Or, the division may occur within the same modality, with attention directed to different locations that are spatially separated [4–6]. Such partitioning of attention almost always exacts a penalty; none of the tasks is performed as well as when each is done on its own. Behavioral and neuroimaging data [7,8] show that accuracy and latency suffer when two stimulus sets must be attended simultaneously. This dual-task interference is well known and can have serious consequence in real world situations. For example—as we discuss later—a driver concentrating on the road and other traffic while simultaneously attending to information presented on an augmented reality head-up display (AR-HUD) will likely experience reciprocal interference that could have a negative impact on safety [9].

### Divided/Partitioned Visual Attention

Whether visual attention can be divided and simultaneously allocated to spatially separated locations has long been the subject of experiment, theory, and controversy [4–6,10–13]. Most investigations and theoretical perspectives have examined the case of a single task where the attentional resource must be distributed over two (or more) spatially separated locations. However, visual attention can be divided without involving non-contiguous locations and can involve distinctly different tasks that require attention. When this happens, the visual attentional processes—though clearly distinct—are spatially *commingled* rather than separated.

This commingled division of visual attention has been little studied. While some explorations of visual attention have used commingled stimuli, this aspect has not generally been their prime focus. For example, in studies of inattentional blindness (IB) [14], the stimuli are often commingled; however, the emphasis has not been squarely on how the attentional resource is distributed between the primary and secondary tasks but rather on the factors that promote the so-called “blindness”. Another instance involves attentional boost experiments [15,16]: in a typical paradigm, participants must memorize a series of briefly presented scenes while watching for a target in a random sequence composed of targets (e.g. white squares) and distractors (e.g. black squares) centered on each scene. Memory for scenes that accompany the targets is generally found to be better than for scenes accompanying the distractors. This attentional boost is assumed to result from a generalized processing enhancement associated with the appearance of the target. A further example of commingled sets of stimuli comes from studies of the role of attention in the subitizing and counting ranges during the enumeration of multiple objects. Vetter [17] differentiated to-be-enumerated sets of spots by using both filled and unfilled spots; participants were cued to enumerate one or the other set, or the whole set. Additionally, Vetter and colleagues [18] used a set of targets and distractors commingled with a central stimulus that was required for a primary task.

Studies of inattentional blindness, the attentional boost effect, and the role of attention during enumeration can provide some insight into how visual attention is allocated under dual-task conditions with sets of stimuli that are not spatially separated. However, each of these areas of investigation had a different principal motivation from ours and none was specifically designed to address the more general question of how attention is divided and deployed during the execution of different visual tasks with distinct stimuli that are spatially commingled. There may be other experimental paradigms that have required the commingled division of visual attention but there seems to have been no systematic study of the general phenomenon.

Existing theories of attention that assume limited attentional and perceptual resources may be compatible with situations where visual attention must be allocated to distinct tasks where the stimuli are spatially commingled. According to load theory [19–21], perceptual processing is limited in capacity but operates in an involuntary manner on all the information within that capacity. A demanding primary task may exhaust available capacity, leaving little or no ability to handle sensory inputs unrelated to the task. However, when the load imposed by the attended task is low, surplus processing resources will be involuntarily allocated to processing other sensory inputs. Thus, load theory would predict that focusing attention on a demanding primary task will impair perception of a spatially commingled but unrelated secondary stimulus. Indeed, with a sufficiently high perceptual load in the primary task, processing the primary stimulus will largely exhaust available capacity, even though the secondary stimulus may share the attended location. Thus, processing of the secondary stimulus will be severely impaired. With a lower primary load, any residual processing capacity will automatically be available for processing secondary stimuli.

An alternative to a load model is a biased competition model [22,23]. This class of models differs from load models principally in how the tasks are prioritized. The allocation of attention is determined based on the results of a competition between the tasks. This task rivalry is modulated by both top-down and bottom-up influences and the primary task does not necessarily receive exclusive priority, as it does in a load model. Thus, the nature and magnitude of the reciprocal accuracy and latency effects between two distinct tasks can help decide whether a load model or biased competition model provides a better account of the division of attention with spatially commingled stimuli. Load models that assume sequential processing steps (allocation of attention to the primary task before involuntary processing of secondary stimuli—subject to remaining capacity constraints), predict that the secondary task will be poorly performed under high primary task loads. However, the primary task will be relatively unaffected by the secondary task, since it has first claim on attentional and perceptual resources. Degradation of primary task performance is expected only under very heavy loads. In short, load models predict little or no effect of the secondary task on primary task performance.

A somewhat different pattern is expected by a biased competition model [22,23] where sensory inputs compete for processing resources and cortical representation. The competition will be most intense among stimuli that are spatially commingled and hence have proximate cortical representations. The competition for processing and representation will be subject to a variety of top-down and bottom-up influences; thus, expectation and stimulus features will play major roles. For example, in our experiments described below, instructions to the participants will induce a bias favoring the primary task of enumeration. This top-down influence will likely be strengthened by participants’ incremental learning of secondary stimulus characteristics as the experiment proceeds. Similarly, primary and secondary stimulus features like size, shape, color, texture, spatial location, and numerosity will provide bottom-up cues to bias the competition for processing. Importantly, in contrast to a load model, there is no necessary prioritization of the primary task, even if this has been emphasized in the experimental instructions or boosted by a learned response bias, such as probability matching over trials. Thus, if the secondary task is found to impair performance (speed and accuracy) of the primary task, this will tend to favor a biased competition model.

**Fig 1:**
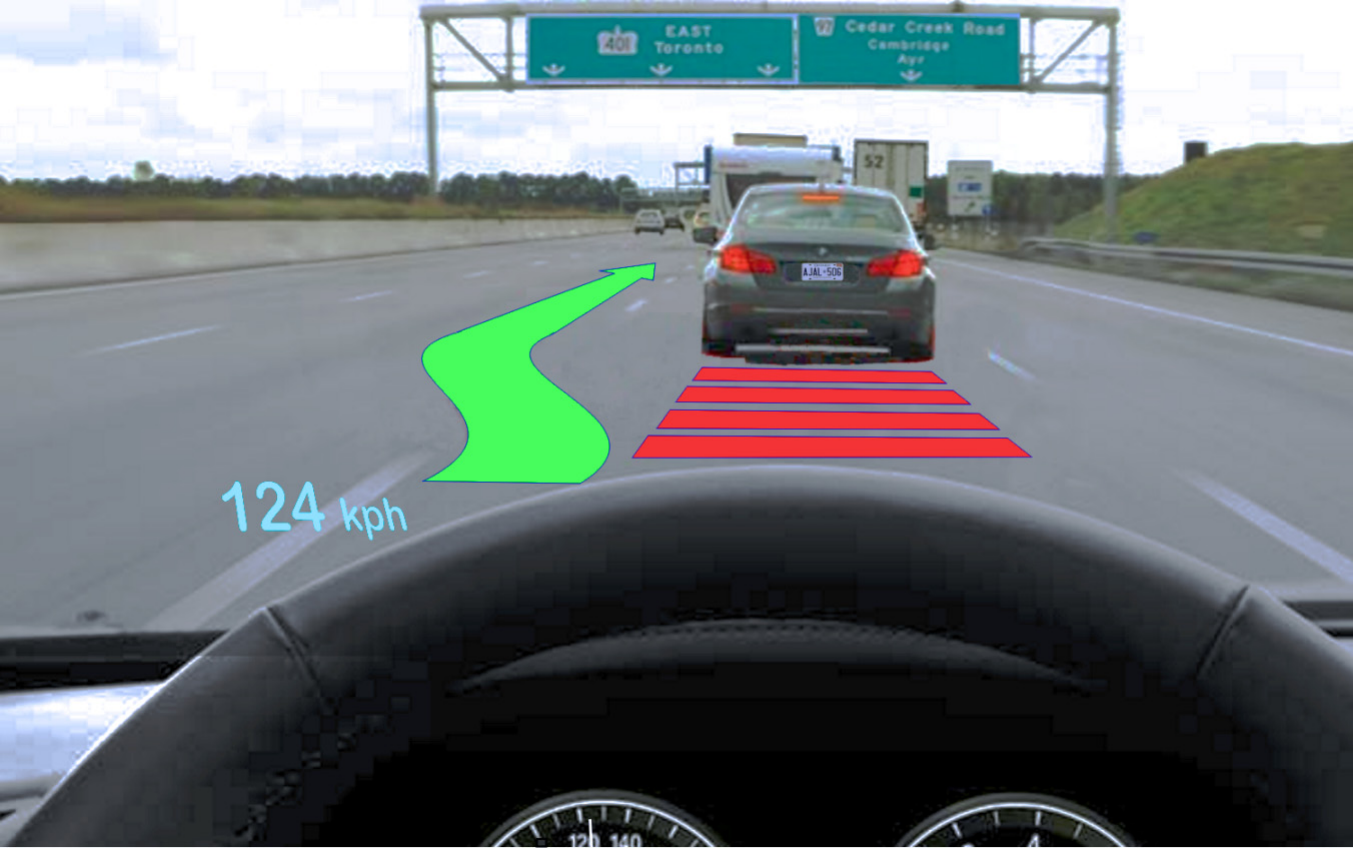
Illustration of an AR-HUD. Using speed, distance, and location, data obtained from remote sensing (cameras, sonar, lidar, GPS, etc.) and map databases, potential threats are evaluated. The slower moving vehicle ahead poses a collision hazard if the red danger zone is entered. Since sensor data confirm no overtaking traffic to the rear, a change in direction is suggested on the AR-HUD. Such augmented reality warnings and suggestions will normally appear infrequently—and often unpredictably—while the driver attends continuously to other tasks that require visual attention (monitoring traffic and road hazards, attending to signage, etc.)

In applied contexts, a better understanding of the fundamental aspects of the commingled division of visual attention may help us to appreciate how new technologies may stretch the capacities of visual attention in ways that could compromise safety. One recent case in point is the augmented reality head-up display (AR-HUD) where computer-generated images are superimposed on the external visual world by projection onto the windshield. Drivers have access to useful information without having to take their eyes off the road. When the driver attends to intermittent graphic warnings on an AR-HUD, while concurrently attending to potential threats beyond the windshield, the attended spatial locations are commingled. Fig 1 shows a hypothetical scenario where an AR-HUD warns of a potential threat and suggests a change in direction to the driver. However, a variety of technical, perceptual, and cognitive challenges could compromise safety and utility [9]. Laboratory investigation of the visual attentional processes involved can provide a valuable perspective. Results from controlled lab trials can establish whether relevant fundamental capacities are likely to be compromised by this variety of divided visual attention.

In two experiments, we had observers repeatedly perform a primary task requiring the deployment of attention over a fairly wide field of view, similar in size to that encountered while driving. At unpredictable intervals, the observer had to perform an additional task that required the detection or identification of a distinctly different stimulus that was spatially commingled with the stimuli of the primary task. The secondary stimulus was initially unexpected, and thus, the first set of trials (only) mirrored the classic inattentional blindness (IB) paradigm. Thereafter, during hundreds of subsequent trials, the secondary stimulus was not completely unexpected, but the probability of its appearance was relatively low.

### The Classic IB Paradigm

In the classic IB paradigm [14,24–26], on each of a small number of *initial trials*, the observer performs a primary task requiring attention (e.g., estimating the number of spots in a display). On a single subsequent *inattention trial*, an additional stimulus (e.g. a square) unexpectedly appears. Observers often fail to report the novelty even though it is in plain view. The phenomenon was probably first documented by Jevons [27] and has since been studied by several authors [28–30]. But it was Mack and Rock who coined the memorable term *inattentional blindness* (IB) to describe this failure [14]. However, their charming label is somewhat misleading since not all observers fail to notice the unexpected stimulus.

### The Iterated Paradigm

The classic IB design cannot shed light on the interplay between expectation and the attentional loads imposed by the primary and secondary tasks. Hence, our experiments extended the basic paradigm by repeating—at unpredictable intervals—the appearance of the secondary stimulus, over the course of hundreds of trials, while simultaneously varying the attentional load imposed by the primary task. Thus, our interest was not in the initial classic IB trial, but rather in its repetition when the observer was aware that an additional stimulus *might* appear during performance of the primary task. The repetition allows assessment of performance under varying levels of load on the visual attentional system and allows the reciprocal effects of the primary and secondary tasks to be quantified. The iterated primary task may be considered to be an experimental analog of the normal allocation of visual attention to road and traffic while driving. The secondary task, where a target shape appears with relatively low probability, is analogous to the appearance of a visual warning in an augmented reality display.

Our first experiment examined the observer’s ability to detect the presence of a partially expected secondary stimulus while simultaneously performing a primary task that required varying amounts of attention. Our second experiment required *identification* of the secondary stimulus, thus ensuring that the stimulus had been clearly seen and was available for further perceptual/cognitive processing.

### Human Participant Research

This research involving human participants was approved by the Office of Research Ethics, Office of the Vice-President, Research and Innovation, University of Toronto (Protocol Reference # 22139) and was conducted according to the principles expressed in the Declaration of Helsinki. Informed consent, both written and oral, was obtained from each participant for both experiments in the study.

## Experiment 1

The primary task was to estimate how many black spots (1, 2, 3, 4, 5, 7, or 8) had been briefly presented on a computer display. Enumeration of sets of objects has long been known to require the allocation of visual spatial attention [27,31], and even with fewer than four objects—the so-called subitizing range—attention is required [17,18,32]. The secondary task required detection of a single outline square that was spatially commingled with the spots. This distinct shape was unexpected on the first trial but was at least partially expected thereafter.

### Materials and Methods

#### Participants

Undergraduate students (23 males, 22 females, mean age 19.0 years) at the University of Toronto participated for course credit. They were recruited from an introductory psychology class and were naïve as to the real purpose of the experiment. In order to encourage them to concentrate on the primary task, participants were told that the three best performers in reporting the number of spots (accuracy and latency, weighted equally) would win $50, $30, and $20 respectively. Informed written consent was obtained from all participants.

#### Stimuli

Stimuli were displayed on a 21-inch Viewsonic Professional Series CRT monitor using E-Prime 2.0 [33] on a gray rectangular background (RGB: 124,124,124; approximately 36° x 27°, width x height). The stimuli for the primary (enumeration) task were filled black circular spots (RGB fill: 0,0,0; each approximately 1° in diameter; 1, 2, 3, 4, 5, 7, or 8 in number; note that 6 spots never appeared) and the unexpected object was a black-outlined shape (a square subtending approximately 2°; RGB outline: 0,0,0; RGB fill:124,124,124). The unexpected shape was fully visible and clearly distinct from the spots, in size, in shape, and in fill. The positions of the spots were distributed randomly within the 36° x 27° rectangular area and no pair was permitted to be closer than 2°. The shape also appeared at a random location within the 36° x 27° rectangular area, no closer than 2° to any of the spots.

Viewing distance was controlled by the use of a combined chin and forehead rest (University of Houston, College of Optometry, HeadSpot). The center of the screen was positioned in the mid-sagittal plane at eye height at a distance of 30 cm. Participants responded using the numeric keypad on a computer keyboard.

#### Procedure

**Fig 2:**
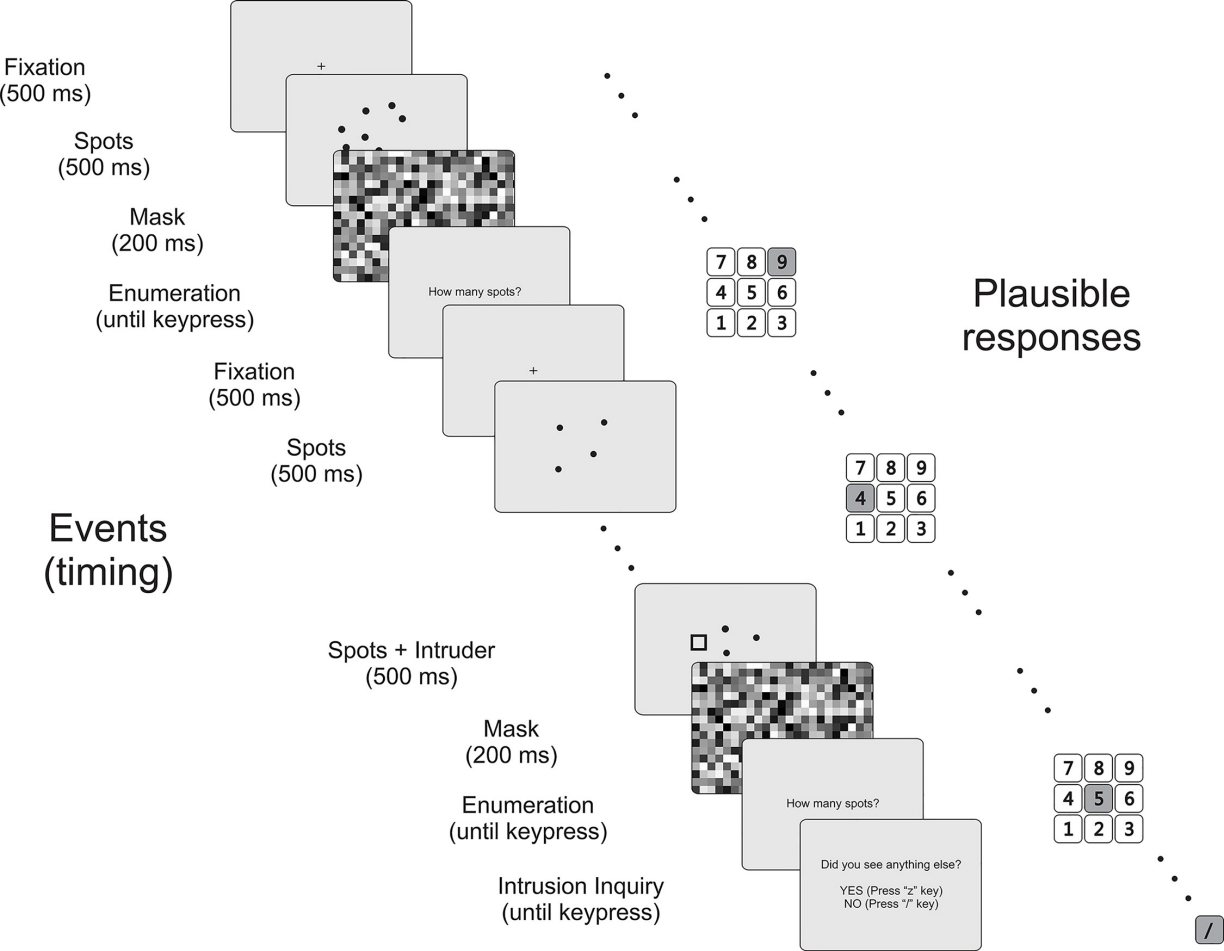
Sample trial sequence with two tasks. Enumeration (primary task) and reporting a shape appearing in addition to the spots (secondary task). Illustrative responses are shown by shading the chosen key on the numeric keypad. In addition to 7 practice and 7 initial trials, there was one classic IB trial (with an unexpected shape); 486 shape-absent/shape-present trials, distributed at random; and 9 full-attention trials. The number of spots (1, 2, 3, 4, 5, 7, or 8) varied randomly with equal probability.

Participants were asked to report the number of spots (primary task: enumeration) on each trial and to respond as quickly and accurately as possible when prompted. Each trial began with a centrally positioned cross (1° x 1°; 500 ms) to establish fixation, followed by a display (36° x 27°; 125 ms) containing a number of spots, (1, 2, 3, 4, 5, 7, or 8), randomly chosen with equal probability; in addition, a secondary stimulus appeared on some trials. The exposure time (100 ms) was short enough to preclude an eye movement. The secondary stimulus was an unfilled black-outlined square. The display was then masked (500 ms) using a randomly pixelated screen (Fig 2).

#### Classic IB Trial

Participants completed seven practice enumeration trials to familiarize themselves with the response keys and the experimental procedures. The practice trials preceded and were identical to seven *initial trials*, which followed immediately. After each initial trial, participants saw the prompt: “How many spots?” On the 8th trial, in addition to the spots, the shape appeared. After completing the enumeration, the participants were prompted again: “Did you see anything else? Press ‘/’ for Yes and ‘z’ for “No”. The numbers of spots appearing with the shape on trial 8 was either 2, 5, or 8, distributed randomly over the 45 participants, such that each of three subsets of 15 participants experienced only one of the three primary attentional loads (2, 5, or 8 spots). Participants did not know that anything extra or unusual might appear on the eighth experimental trial—the inattention trial—and thus, the appearance of the shape was completely unexpected. Up to this point, the experiment followed the classic inattentional blindness paradigm [14].

#### Iterated Trials

The next 486 trials—the iterated trials—were grouped in 3 blocks of 162 trials. Participants were allowed a short self-paced rest between blocks. On each trial: (1) the number of spots was random with equal probability from the set {1, 2, 3, 4, 5, 7, 8}; (2) the shape could appear with probability.075; and (3) as in the classic IB trial, participants responded to an additional prompt (“Did you see anything else? Press ‘/’ for Yes and ‘z’ for “No”) after each enumeration. The number of *shape-absent* displays intervening between *shape-present* displays could be quite large since the distribution of the number of trials between random equiprobable appearances of the shape is geometric with expectation μ = 12.3 and standard deviation σ = 12.8 [34]. While the shape could conceivably appear on successive trials, a gap of 20 or more trials before its reappearance was also not highly unusual.

#### Full-attention Trials

On the next nine trials, participants were instructed not to report the number of spots but only to say whether the shape, which appeared on all of these trials, was present. The number of spots was 2, 5, or 8, chosen at random. These trials—the full-attention trials—are standard in classic IB experiments and verify that the shape was fully visible when attention was not required for the primary task of enumeration.

### Results

#### Classic IB Trial

The classic IB paradigm is based on a single trial only and is not of major interest here. The proportions of correct detection and a chi-squared test for equivalence of proportions are included mainly for comparison with the existing IB literature. Reporting rates for the three primary task loads in the naïve condition were significantly different, X^2^_(2)_ = 6.14, p<.05: with 2 spots to enumerate, 7 of 15 participants (47%) reported the shape; with 5 spots, 4 of 15 participants (27%) were successful; and with 8 spots, only 1 of the remaining 15 participants (7%) reported the shape.

#### Iterated Trials

For the iterated trials, we computed the proportion of correct responses in each experimental treatment combination. These proportions were transformed using a variance-stabilizing square root-arcsine transformation before conventional repeated measures analysis of variance. Although the statistical tests were calculated on the transformed data, the reported means and graphs use the original proportions for simplicity of interpretation.

#### Primary Task (Enumeration)

**Fig 3:**
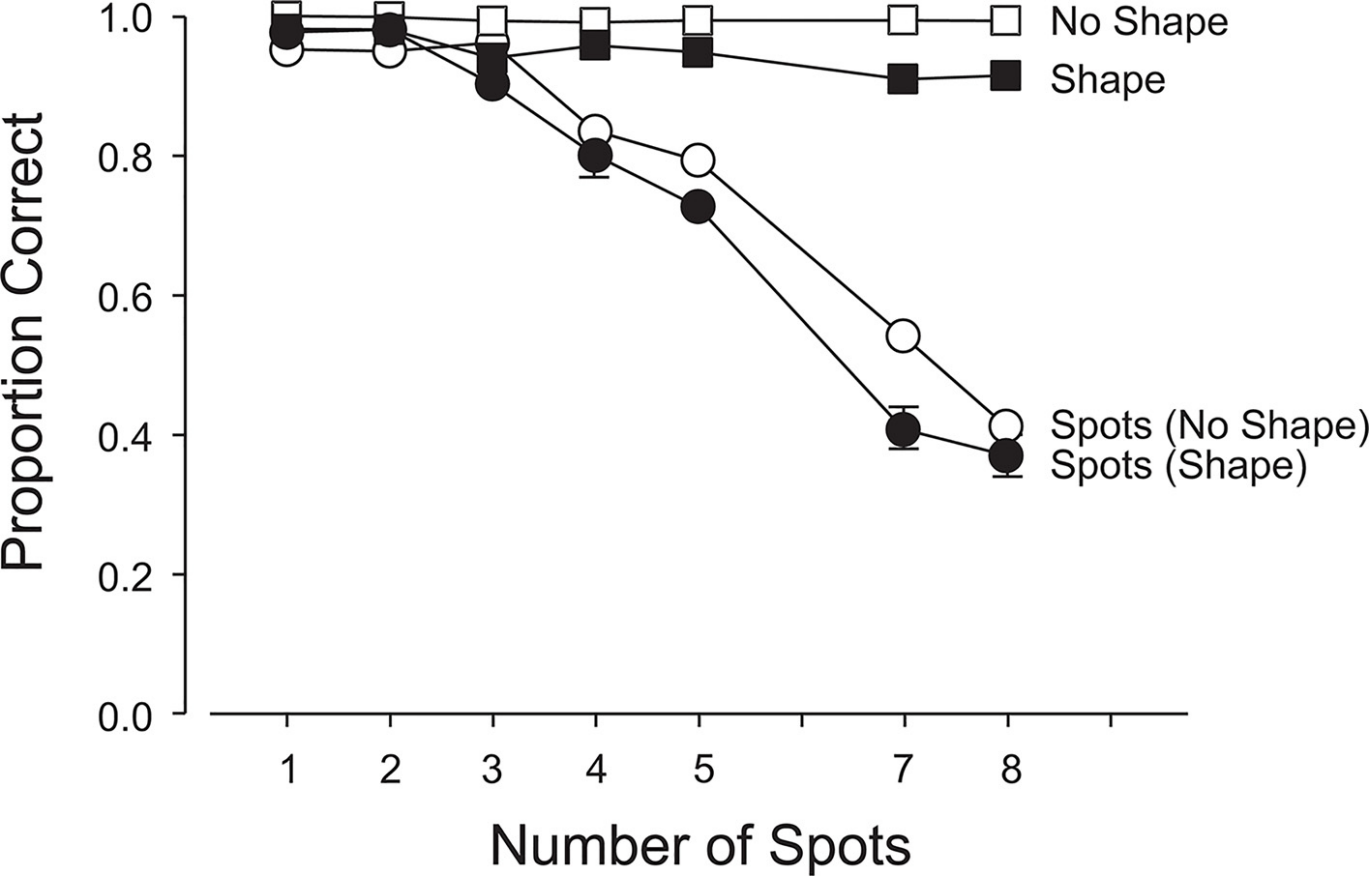
Experiment 1: Accuracies in the primary (enumeration) and secondary (shape-detection) tasks. 1. Open Squares: proportion correct negative responses (1- false alarm rate) when the shape was absent; 2. Filled Squares: proportion correct positive responses (hit rate) when the shape was present; 3. Open Circles: proportion correct enumerations when the shape was absent; and 4. Filled Circles: proportion correct enumerations when the shape was present. The error bars show 1 s.e.m.; if no bars are visible, the 2 s.e.m. range centered on the mean is smaller than the height of the marker. Means associated with open symbols are each based on approximately 2761 data points. Means associated with closed symbols are each based on approximately 224 data points.

**Fig 4:**
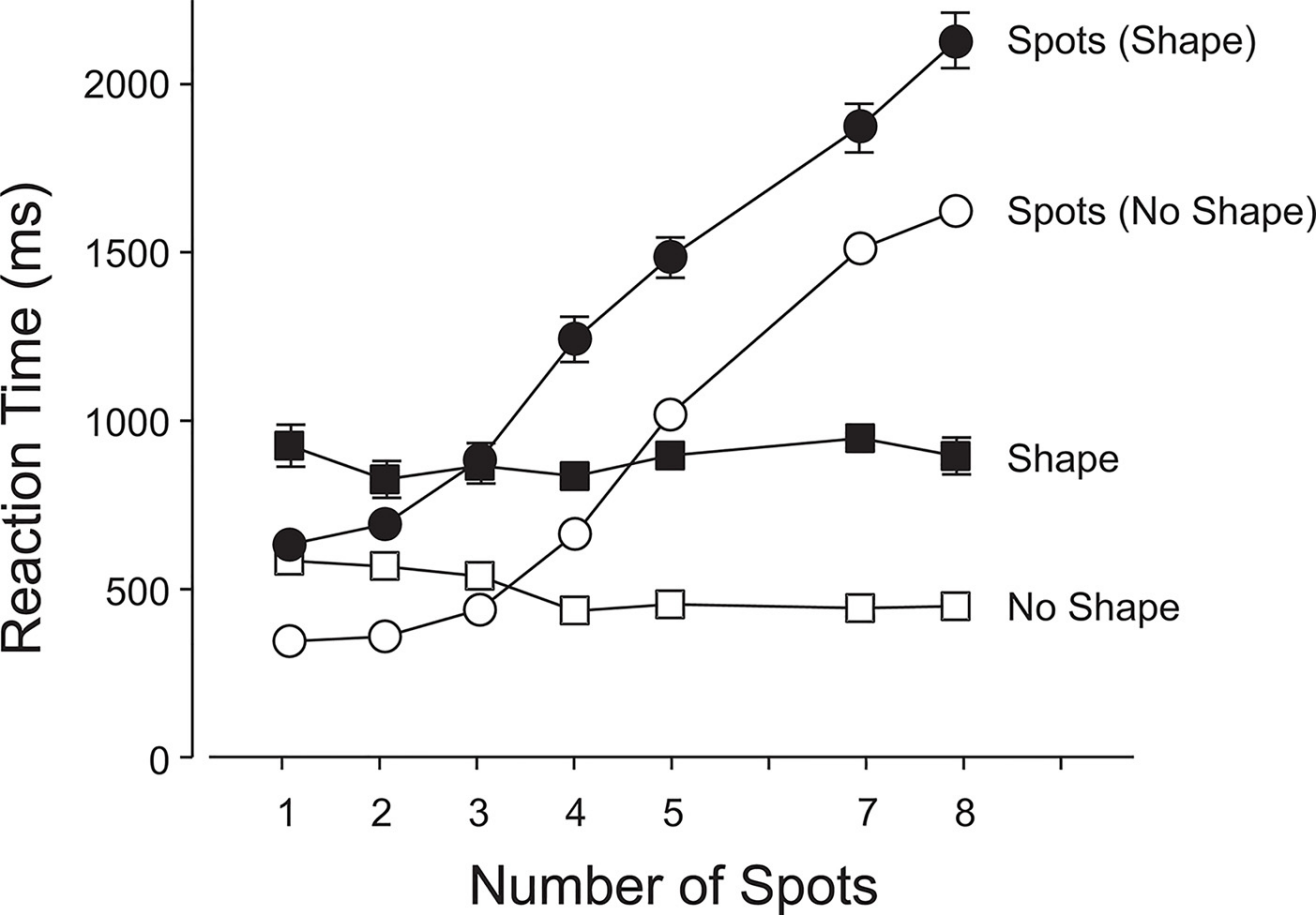
Experiment 1: Latencies in the primary (enumeration) and secondary (shape-detection) tasks. 1. Open Squares: mean reaction times when the shape was absent; 2. Filled Squares: mean reaction times when the shape was present; 3. Open Circles: mean reaction times for enumeration when the shape was absent; and 4. Closed Circles: mean reaction times for the enumerations of the number of spots when the shape was present. The error bars show 1 s.e.m.; if no bars are visible, the 2 s.e.m. range centered on the mean is smaller than the height of the marker. Means associated with open symbols are each based on approximately 2761 data points. Means associated with closed symbols are each based on approximately 224 data points.

Accuracy declined as the number of spots increased (Fig 3) on both shape-present and shape-absent trials, F(6, 264) = 130.70, p< .0001. The anticipated drop off in accuracy (Fig 3) and increase in latencies (Fig 4) beyond the subitizing range of 1–3 spots were observed. On average, both accuracy and latency were better on shape-absent trials than on shape-present trials; 76.4% vs. 72.3%, F(1,44) = 329.97, p<.0001 (Fig 3); 865 ms vs. 1293 ms, F(1,44) = 196.67, p<.0001 (Fig 4). There were small but significant interactions between the type of trial and the number of spots: for accuracy: F(6,264) = 5.73, p<.0001 (linear x linear component: F(1,264) = 20.69, p<.0001; quadratic x quadratic component: F = 5.88, p<.05) and for latency: F(6,264) = 3.00, p<.01 (linear x linear component: F(1,264) = 6.73, p<.0001; quadratic x quadratic component: F = 4.68, p<.05). Figs 3 and 4 show that the accuracies and latencies diverge slightly with an increasing number of spots.

#### Secondary Task (Shape-absent vs. Shape-present Trials)

Participants responded correctly on 98% of the shape-absent trials compared to 93% correct responses on the shape-present trials; F(1,44) = 1067.07, p<.0001 (Fig 3). Mean RTs were 541 ms and 907 ms, respectively; F(1,44) = 101.90, p<.0001 (Fig 4). There was a small but just significant interaction between shape-present and shape-absent trials and the number of spots enumerated; F(6, 264) = 2.19, p<.05. Fig 4 reveals a small difference in slopes, with slightly quicker responses for an increasing number of spots when the shape is absent (linear x linear component: F(1,264) = 4.61, p<.01).

#### Full-attention Trials

Reporting accuracy was 99.8%. The shape was clearly visible among the spots when participants were not required to enumerate them.

### Discussion

On the classic IB trial, when attention was lightly loaded by the primary task, 47% of participants reported the unexpected shape; but when visual attention was more heavily occupied, this percentage dropped to 27% and eventually to only 7%. This replicates previous research on inattentional blindness: participants can detect simple, salient geometric objects with low to moderate probability when they are concurrently engaged in a sufficiently demanding attentional task and are naïve to the possibility of the appearance of an additional distinct visual stimulus [14,24–26,35–37]. Our major interest, however, was in the iterated trials where the participants were aware that a shape might appear in addition to the spots that they had to enumerate.

When the shape was absent, there were virtually no false alarms, independent of load on the primary task (Fig 3), suggesting that very little in the way of attentional resources was required to confirm that the secondary stimulus had not appeared. However, when the primary task imposed a light load (1–4 spots) and the shape appeared, it was missed on about 1 trial in 15 on average. With a heavier primary load (5–8 spots), the miss rate increased to around 1 in 10 trials. This suggests that if attention is increasingly occupied by the primary task, participants will have more difficulty in detecting the secondary task stimulus. Nonetheless, even with heavy primary task loads, the shape was detected with fairly high probability. The latency to respond (in the secondary task) was 907 ms when the shape was present and 541 ms when it was absent (see Fig 4). These results tend to favor a biased competition account of the division of attentional resources over a load model since it appears that the primary task was not sufficiently prioritized over the secondary task to severely impair processing of the latter. The increase in RT when the shape was present is likely a consequence of the competition for attention and representation between the two commingled stimuli. It simply takes longer to divide and allocate attention when both stimuli are competing for resources. However, the faster response on shape-absent trials may—in part—be the result of a bias to respond negatively, due to the preponderance of shape-absent trials.

With no shape present, the primary task accuracy decreased from just over 90% (1 spot) to about 40% (8 spots) with an elbow at 3 spots (Fig 3). This confirms that increasing the task load in enumeration has a negative effect on performance and is also consistent with the well-known difference between subitizing and counting. With the shape present, primary task accuracy was little affected with light primary task loads (1–4 spots), but for heavier loads (5–8 spots), the primary task accuracy was about 7% worse on average (see Fig 3 and the significant linear x linear component of the interaction). Costs are incurred with the deployment of visual attention to detect an intruding shape. When the primary task is easy, the secondary task is accommodated without discernable cost, but as the primary task becomes more demanding, both tasks suffer reciprocal interference, suggesting a competition for attentional resources rather than a prioritized allocation, with the primary task dominant.

In many applied situations—such as driving a vehicle—it would not be sufficient to merely detect the appearance of a relatively unexpected secondary stimulus. Identification is likely to be required. Attention must be deployed and maintained for the observer to be able to select the response from a set of (partially) expected options. For example, in an automotive augmented reality display, it would be important to distinguish images warning of (1) a forward collision from (2) a lane excursion or (3) an imminent turn advisory. In AR displays, visualizations of threats that are not clearly distinguishable are likely to be more dangerous than no warning at all. Thus, experiment 2 included the additional task of identification.

## Experiment 2

As before, the primary task was enumeration of a number of black spots. The secondary task outline shape was a triangle, a square, or a diamond. Each of these three variants appeared an equal number of times during the iterated trials. The participants had to detect and identify the shape that had appeared, thus ensuring that it had been clearly seen.

### Materials and Methods

#### Participants

Forty-five different undergraduate student volunteers (22 males, 23 females, mean age 19.4 years) participated under the same conditions as in experiment 1. Informed written consent was obtained from all participants.

#### Apparatus

The same apparatus was used as in experiment 1.

#### Stimuli

In order to extend the range of enumeration, the number of spots was 3, 4, 5, 6, 7, 8, or 9. The identity of the secondary task stimulus (triangle, square, or diamond) was randomly determined subject to the restriction that each shape appeared the same number of times. Otherwise, the stimuli and conditions were the same as in experiment 1.

#### Procedure

This was the same as in experiment 1 with one exception. Instead of the question, “Did you see anything else?” participants were asked “Did you see any of the following: 1. triangle; 2. square; 3. diamond; 4. none of these? Press the appropriate number key.” This prompt occurred on *every* iterated trial—after the enumeration—whether a shape had appeared or not.

### Results

#### Classic IB Trial

The classic IB paradigm is based on a single trial only and is not of major interest here. The proportions of correct detection and a chi-squared test for equivalence of proportions are included mainly for comparison with the existing IB literature. When the shape (triangle, square, or diamond) was not expected, the identification rates varied with the attentional load on the primary task; X^2^_(2)_ = 5.83, p = .05. With 3 spots to enumerate, 6 of 15 participants (40%) correctly identified the shape; with 6 spots, just 2 of 15 different participants (13%) were successful; and with 9 spots, only 1 of the remaining 15 participants (7%) identified the shape.

#### Iterated Trials

We computed the proportion of correct responses in each experimental treatment combination. These proportions were transformed using a variance-stabilizing square root-arcsine transformation before conventional repeated measures analysis of variance. Although the statistical tests were calculated on the transformed data, the reported means and graphs use the original proportions for simplicity of interpretation.

#### Primary Task (Enumeration)

**Fig 5:**
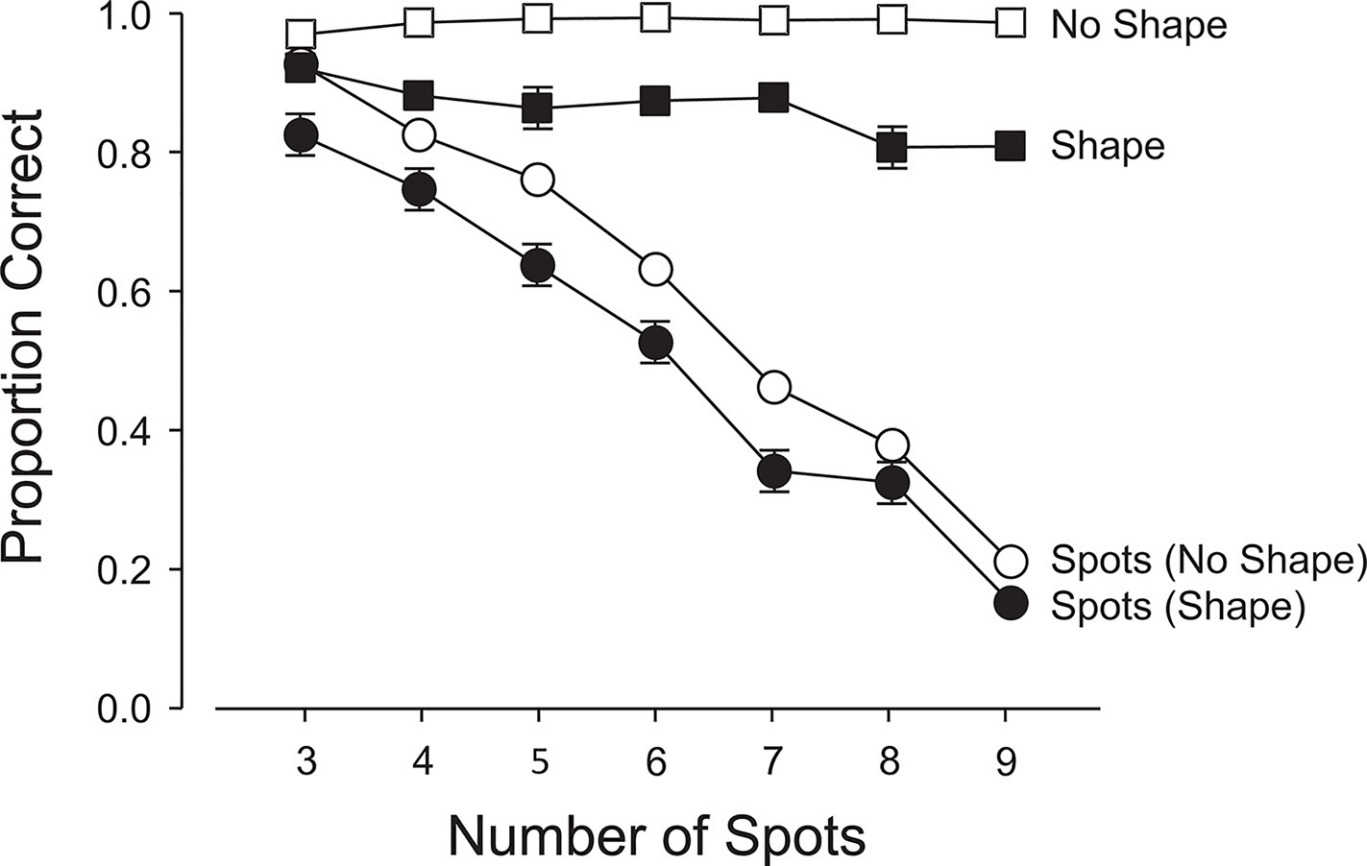
Experiment 2: Accuracies in the primary (enumeration) and secondary (shape-detection) tasks. 1. Open Squares: proportion correct negative responses (1- false alarm rate) when the shape was absent; 2. Filled Squares: proportion correct positive responses (hit rate) when the shape was present; 3. Open Circles: proportion correct enumerations when the shape was absent; and 4. Filled Circles: proportion correct enumerations when the shape was present. The error bars show 1 s.e.m.; if no bars are visible, the 2 s.e.m. range centered on the mean is smaller than the height of the marker. Means associated with open symbols are each based on approximately 2761 data points. Means associated with closed symbols are each based on approximately 224 data points.

**Fig 6:**
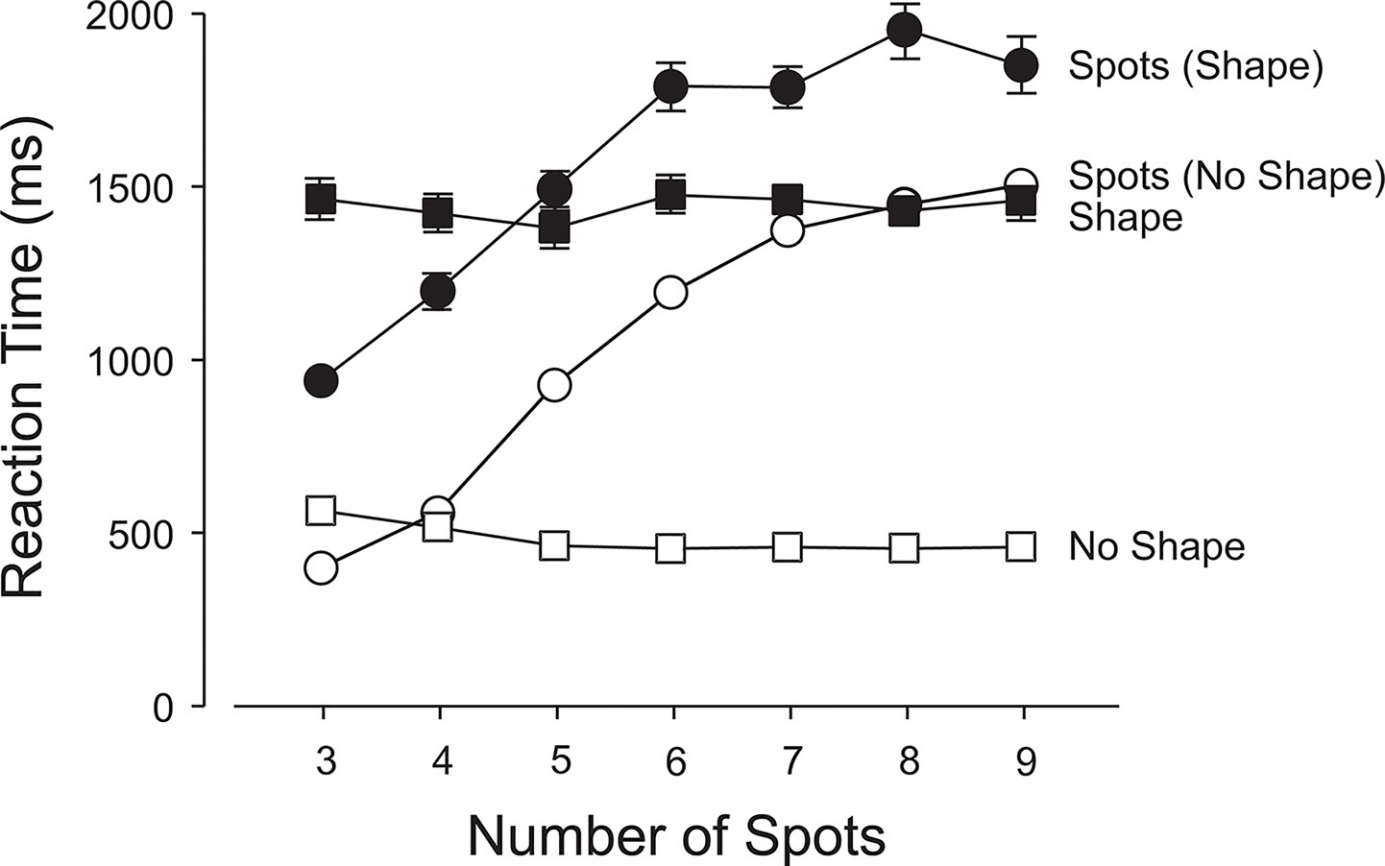
Experiment 2: Latencies in the primary (enumeration) and secondary (shape-detection) tasks. 1. Open Squares: mean reaction times when the shape was absent; 2. Filled Squares: mean reaction times when the shape was present; 3. Open Circles: mean reaction times for enumeration when the shape was absent; and 4. Closed Circles: mean reaction times for the enumerations of the number of spots when the shape was present. The error bars show 1 s.e.m.; if no bars are visible, the 2 s.e.m. range centered on the mean is smaller than the height of the marker. Means associated with open symbols are each based on approximately 2761 data points. Means associated with closed symbols are each based on approximately 224 data points.

As expected [32], accuracy of enumeration declined as the number of spots was increased on both shape-present and shape-absent trials; F(6, 264) = 132.07, p<.0001 (Fig 5). On average, accuracy of enumeration was better on shape-absent trials than on shape-present trials (60% vs. 50%), F(1, 44) = 189.00, p<.0001 (Fig 5), and latencies on the shape-absent trials were lower than on shape-present trials (1071 ms vs. 1588 ms), F(1, 44) = 198.98, p<.0001 (Fig 6). There was a small but significant interaction between the type of trial and the number of spots, F(6, 264) = 3.78, p<.01, and Fig 6 reveals a slight reduction in the shape-identification latencies on shape-present (relative to shape-absent) trials with an increasing number of spots (linear x linear component, F(1,264) = 14.50, p<.001).

#### Secondary Task (Shape-absent vs. Shape-present Trials)

Participants responded correctly on 99% of the shape-absent trials compared to 86% correct responses on the shape-present trials, F(1,44) = 1002.19, p<.0001 (Fig 5). Respective mean RTs were 493 ms and 1451 ms, F(1,44) = 402.30, p<.0001 (Fig 6). The primary task attentional load had a significant effect when the shape was present: as the number of spots increased from 3 to 9, the reporting accuracy of the shape fell from 92% to 81%; F(6, 264) = 4.52, p<.001 (Fig 5). Accuracy in identifying the different shapes differed, F(2, 44) = 7.27, p<.01, with the diamond significantly poorer (81%) than the triangle (89%) or square (88%), which did not differ significantly from each other (Student-Newman-Keuls, p = .05; critical ranges: 4.8%, 4.0%).

#### Full-attention Trials

Identification accuracy was 95%. The shape was correctly identified when participants were not required to enumerate the spots, and they missed only 1 trial in 20 on average.

## Discussion

Experiment 2 replicated experiment 1 in all important respects and extended the range of the number of spots to be enumerated in the two experiments to 1 through 9. Whereas participants in experiment 1 did not have to identify the shape when it appeared, the new participants in experiment 2 had to perform the extra task of identifying the particular shape (triangle, square, or diamond). During the classic IB trial, when attention was lightly loaded, 40% of participants successfully identified the unexpected shape; but when visual attention was heavily occupied, only 7% of participants succeeded. Although the probability of identification was reduced with an increasing attentional load in the primary task, it seems that the available attentional resource was shared between the two distinct visual objects (configuration of spots vs. outline shape), even though participants were initially unaware that an additional object would appear.

During the iterated trials, when participants were no longer naïve, they reported and identified the secondary stimulus between 81% and 92% of the time depending on the magnitude of the load in the primary task. Participants were less likely to identify the shape with heavier loads in the primary task, although even at the highest load (9 spots), the shape was identified in more than 4 trials out of 5. When the shape appeared, the average time to respond was 1413 ms; when it was absent, the average was 484 ms. Since participants not only had to notice the shape but also to report its type (triangle, square, or diamond), choosing the correct shape increased the latency of the response over a simple present-absent judgment. The time taken for enumeration was also greater when a shape was present than when it was absent. These latencies are consistent with a reduction in the share of the attentional resource allocated to the primary task when a shape appeared. Division of the attentional resource seems to have been fine tuned in real time depending on whether one object (the configuration of spots) or two objects (the configuration of spots plus the shape) had appeared. The appearance of the partially expected shape had a reciprocal effect on performance of the primary task. Similarly, increasing the attentional load in the primary task reduced the probability of correctly identifying the shape when it appeared. These findings tend to favor a biased competition model over a load model of the division of attentional and perceptual processing resources.

The overall success rate in the full-attention trials dropped from almost 100% in experiment 1 to 95% in experiment 2. The corresponding drop for iterated trials was from 93% to 86%. The additional requirement to identify the shape accounts for the drop in accuracy. Furthermore, the difference in average latency between the iterated trials in the two experiments was about 500 ms, reflecting the time required to identify the shape. Identification imposes additional costs in speed and accuracy.

## General Discussion

The classic IB trial in both experiments replicated previous research on inattentional blindness [14,24–26]. Although there was only one task—from the participant’s point of view—the available visual attentional resource was, in fact, divided between the two tasks when the unexpected shape appeared. We may infer this since the unexpected shape was frequently detected under low (2 or 3 spots) to moderate loads (5 or 6 spots) in the primary task. It was only likely to be missed if the primary task was very demanding (8 or 9 spots).

When the shape was partially expected, the allocation of attentional resources was strongly influenced by this expectation. Figs 3 and 5 show that the shape was reported between 90% and 97% of the time with a light load (2 or 3 spots) in the primary task. As the attentional load in the primary task increased, the detection or identification rate dropped—but never below 80%. The probability of detection or identification of the shape was inversely proportional to the attentional load in the primary task. Attention was divided in response to the demands of the primary task, just as in the unexpected case (classic IB). The major difference was that partial expectation of the shape (and its perceptual features) strongly modulated the division of attention, resulting in an increased allocation to the shape when it was present. Detection or identification rates of the shape were above 80% at the highest primary task loads while they were as low as 7% in the classic IB trials. Thus, (partial) expectation strongly affected the distribution of attention and was the critical variable in boosting the probability of noticing and identifying the shape.

Allocation of a greater fraction of the attentional resource to a partially expected shape had a reciprocal impact on the primary task. Figs 3 and 5 show that the probability of a correct enumeration was at least 5% lower when a shape was present; and if identification was required, the reduction in accuracy was around 10%. These indications of bias in the allocation of the attentional resource are mirrored in corresponding changes in the response latencies (Figs 4 and 6). Our results confirm that when a limited visual attentional resource must be shared between two spatially commingled visual tasks, both will suffer the adverse effects of dual-task interference. The reciprocal interference will vary in proportion to the demands of the two tasks and will be modulated by the expectations of the observer.

As previously noted, Vetter’s experiments on the attentional mechanisms of enumeration were similar to ours in some respects. Her experiment 1 [17] required participants to enumerate a set of briefly presented dots; some dots were white and the others were black. In separate blocks of trials, participants had to enumerate: (1) all dots (white and black) in the control condition; (2) either the white or the black dots in a pre-cue condition; and (3) either the white or the black dots in a post-cue condition. In the pre-cue condition, participants knew—just before stimulus presentation—whether to enumerate the white or the black spots. In the post-cue condition, they were instructed just after the stimulus was presented. Matching for set size, accuracies for both pre-cued and post-cued numbers of spots were lower than in the control condition, with the post-cued condition yielding the lowest accuracies of the three conditions. Although the black and white dots were commingled in the control condition, this was essentially a single enumeration task. Similarly, the pre-cued condition may be viewed as a single task with distractors (dots of the non-cued color); participants knew which set to prioritize. In the post-cued condition, however, they would have had to guess which set should be enumerated or attempt to enumerate both sets and hold the results in working memory. In the post-cued condition, it is possible that attention was distributed between two sets of commingled stimuli by at least some participants, depending on their chosen strategy (single or attempted dual task). Vetter’s tasks also differ from ours in that the same judgment (enumeration) was required of the participant, whereas our participants made two different judgments (enumeration and detection/identification). Nonetheless, her results are similar to ours in one important respect: the primary task was subject to interference from the non-enumerated stimuli in the pre- and post-cued conditions.

Although attentional boost paradigms [15,16] share the important characteristic of spatially commingled stimuli, there are crucial differences in task demands. The occasional target in the primary task of a typical attentional boost experiment was a white square, and the distractor was a black square; the square appeared superimposed on a to-be-remembered scene at the center of *every* image. The square (either white or black) was always present and there was no spatial variability in its location. The only unpredictable element was whether the square would be white or black. Thus, the attentional demands of the primary task would have been very low. It is not clear which locations in the scene were attended during the secondary task of encoding the scene, but it is likely that the secondary task imposed considerably heavier attentional demands than the task of target detection. Swallow and Jiang [38] have suggested that the attentional boost may be partly a consequence of a very low primary task load. When their target detection task was made more demanding—by introducing a response-mapping task or instructing participants to make arbitrary responses to targets—the attentional boost was eliminated. In our experiments, no attentional boost was observed, perhaps suggesting that our primary task imposed too high a load, thus precluding an attentional boost effect.

### Perceptual Load vs. Biased Competition Models

Which model provides a better account of our results? Perhaps the most important differentiating feature of the two models is how the tasks are prioritized. A load model presumes that processing resources are involuntarily allocated to the secondary task only after the demands of the primary task have been satisfied (at least in high primary task load conditions). The primary task in our experiments was highly prioritized: (1) participants knew that the top three performers in accurately and quickly reporting the number of spots would be awarded $50, $30, and $20; (2) participants performed the primary task on *every* trial; and (3) the secondary stimulus appeared, on average, only once in about 12 trials, thus encouraging a response bias toward the primary task. However, this bias would likely have been somewhat attenuated since participants were prompted after every trial to report a possible secondary stimulus. Nonetheless, it is reasonable to assume that the primary task of enumeration would have been prioritized. Load models predict that performance on the primary task should not differ much whether the secondary stimulus is present or not, since the primary task has priority. Furthermore, under increasing loads in the primary task, the secondary task should be performed more poorly, since less of the processing resource remains after the demands of the primary task have been satisfied. However, our data show that this was not the case in either experiment. When the shape was present, both primary (enumeration) and secondary (detection/identification) tasks experienced losses in accuracy and increased latency.

In contrast, a biased competition model assumes that the allocation of attention to separate tasks is essentially simultaneous; there is a competition among the commingled stimuli for access to processing resources and cortical representation. This contest is biased by a number of factors, including bottom-up features, such as stimulus salience, and top-down influences, such as expectation. Thus, even with a highly loaded primary task, low-level competitive interactions and expectation biases may be enough to tip the balance toward the secondary task. The inevitable result is diminished performance (accuracy, latency) in the primary task compared to when it is the only task. Overall, our data are better described by a biased competition model than a load model. Large reciprocal effects in speed and accuracy were observed when the secondary stimulus (the shape) appeared. Thus, we believe that the allocation of perceptual resources to our primary and secondary task events was negotiated according to a blend of top-down bias based on expectation and bottom-up competitive interactions based on stimulus characteristics.

### Augmented Reality Displays

The essence of augmented reality (AR) is to integrate information with the driver’s forward view—generally in pictorial format—to enhance and draw attention to visual features in the environment. Navigation directions [39], traffic information, weather and road conditions, and imminent collision dangers are communicated to the driver by projecting graphics onto the windshield. The intent is to provide predictions and warnings while reducing driver workload and enhancing occupant safety, as well as that of other road users [9]. AR systems are in development at all major vehicle manufacturers and are being studied in academic settings [40,41]. As AR features are incorporated, the observer’s view becomes more complex. Changes in the visual field will trigger a commingled division of visual attention that may cause the driver to miss sudden threats on the road. The problem is analogous to the use of mobile phones in vehicles where the distraction is auditory (speech) and the impact is on visual attention [1,42].

In the real world of driving, the reciprocal interference between two spatially commingled tasks is likely to be worse than we observed. Our participants reported the presence or absence of the shape on *every* iterated trial; this requirement probably shifted the allocation of attention more toward the possibility of an intruder than if they had not been prompted. In the driving environment, there would be no such prompts and hence the division of attention would probably be biased much more toward the primary task of monitoring the road and traffic. Thus, it is likely that a driver’s expectation of the appearance of an AR warning would generally be lower than that of our participants, who were constantly reminded of the possible appearance of a shape by the prompt on the previous trial. Even if the shape had not appeared for several trials, our participants would have been more alert to the possibility of its appearance than a driver would generally expect an AR warning on the HUD.

Our results suggest that AR-HUD technology may have the following unintended effects: (1) the (partial) expectation of HUD warnings will result in diversion of attention from events and objects in the real world with a loss of accuracy and increase in reaction times in attending to the external information; (2) identification of HUD warnings will be slowed and will be less accurate depending on the attentional load imposed by objects and events in the real world. Paradoxically, it is possible that the capture of attention by AR warnings may result in the driver paying less attention to visual threats in the real—as opposed to virtual—world. It is precisely when the attentional load imposed by real-world events is increased that the HUD is likely to generate AR warnings. But because of the increased level of external threats (and the consequent demands on visual attention), the HUD warnings will be more likely to be missed or misidentified.

AR-HUD displays bring with them new and, as yet, poorly understood challenges to driver safety [9]; laboratory experimentation to establish the limits of human capacities in deploying attention to commingled—but distinct—visual events is necessary. Simulator experience [43] and on-road testing [44] will be critical in revealing potential problems; and epidemiological data [1] will be even more informative. But until such data are available, laboratory experiments that mimic the cognitive demands of real-world driving may provide the best indication of potential threats to safety.

### Conclusion

When visual attention must be divided and allocated to distinct but spatially commingled stimuli, participants’ performance is compatible with a biased competition model [22,23]. A perceptual load model [21] would predict that a sufficiently demanding primary task will exhaust processing resources, leaving little or nothing for the secondary task. In a biased competition account, attention would be allocated competitively and simultaneously, implying that the secondary task might hijack resources that could have been allocated to the primary task. Our data are compatible with a biased competition account since our secondary task had a clear impact on performance of the primary task in terms of accuracy and processing time.

When driving, an AR-HUD warning is most likely to occur in a high load condition, such as that encountered in heavy traffic. Our data suggest that the AR-HUD warning may have an unexpected and unintended consequence—the driver’s attention to the road and traffic is likely to be compromised due to the competition between the normal perceptual processing associated with attending to the road and the need to allocate attentional and other cognitive resources to dealing with the AR-HUD warnings. Furthermore, this rivalry for the driver’s attention is most likely to occur when the driving environment is demanding.
